# The production of tyramine *via* the selective hydrogenation of 4-hydroxybenzyl cyanide over a carbon-supported palladium catalyst†

**DOI:** 10.1039/c8ra05654d

**Published:** 2018-08-20

**Authors:** Mairi I. McAllister, Cédric Boulho, Liam McMillan, Lauren F. Gilpin, Sandra Wiedbrauk, Colin Brennan, David Lennon

**Affiliations:** School of Chemistry, University of Glasgow Joseph Black Building Glasgow G12 8QQ UK David.Lennon@glasgow.ac.uk +44-141-330-4372; Syngenta, Jeallot's Hill International Research Centre Bracknell, Berkshire RG42 6EY UK

## Abstract

The selective production of primary amines is a problem that plagues heterogeneously catalysed nitrile hydrogenation reactions. Whilst the target amine tyramine (HOC_6_H_4_CH_2_CH_2_NH_2_) is biochemically available through the action of enzymes, synthetic routes to this species are not widely reported. Here, a heterogeneously catalysed method is proposed that utilises a Pd/C catalyst to effect the selective hydrogenation of 4-hydroxybenzyl cyanide within a three-phase reactor. The aforementioned selectivity issues are overcome by adjustment of various experimental parameters (hydrogen supply, agitation rate, temperature, use of an auxiliary agent) that result in improved catalytic performance, such that the desired tyramine salt (tyramine hydrogen sulphate) can be produced in quantitative yield. Accordingly, through consideration of the interconnectivity of hydrogenation and hydrogenolysis processes, a selective synthetic strategy is achieved with the findings suitable for extension to other substrates of this nature.

## Abstract 2

Tyramine hydrogen sulphate is produced *via* the heterogeneously catalysed selective hydrogenation of 4-hydroxybenzyl cyanide within a three-phase reactor.

## Graphical abstract

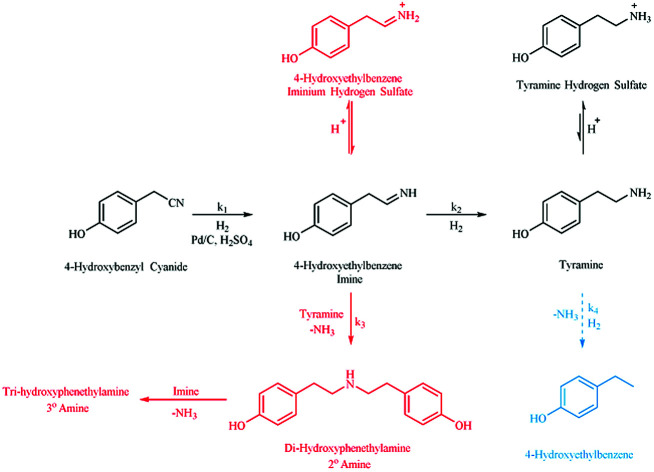

## 1. Introduction

Primary amines are chemically significant reagents finding application in many industries including pharmaceuticals, plastics and agri-chemicals.^1^ Tyramine (HOC_6_H_4_CH_2_CH_2_NH_2_) is such a species and is a molecule which presents considerable interest due to its biological activity in the human body, where it may be accessed *via* foods as diverse as cheese, red wine, sausages and chocolate.^2^ In addition to involvement in a range of biochemical pathways within mammalians, trace amines such as tyramine and its analogues are additionally thought to influence aspects of brain chemistry. Biochemically, tyramine may be produced by the action of the enzyme tyrosine hydroxylase to form tyrosine (HOC_6_H_5_CH_2_CH(CO_2_H)(NH_2_)) that, subsequently, undergoes decarboxylation *via* the action of the enzyme tyrosine decarboxylase to form tyramine. The matter of the pharmacology and therapeutics of trace amines has been comprehensively reviewed by Broadley.^2^

The value and biochemical availability of tyramine has been demonstrated, and thus, development of a chemical synthetic strategy is desirable. There are a number of different ways in which primary amines can be synthesised including alkylation with ammonia and the reductive amination of oxo compounds,^3^ as well as *via* the heterogeneously catalysed hydrogenation of nitriles.^4–7^ It is the later option which is explored herewith. In the case of nitrile hydrogenations, conversion of the nitrile functionality is a facile process,^8^ nonetheless, achieving high selectivity towards the primary amine is significantly more challenging due to the presence of a highly reactive imine intermediate. Amongst other side reactions, condensation reactions between this species and the amine product, in a process initially proposed by Braun *et al.*,^9^ can lead to the formation of undesired side products such as secondary and tertiary amines and, consequently, a reduced selectivity to the desired product.^5,10,11^

Whilst previous investigations into the selective hydrogenation of aromatic nitriles in the liquid phase have highlighted the use of skeletal metal catalysts such as RANEY® Cobalt,^12^ application of supported Pd catalysts has increasingly featured in recent years, not least because Pd is thought to minimise reduction of substituent groups, other than the nitrile functionality, when present. This attribute is particularly pertinent to fine chemical applications. Notable examples being the work of Hegedűs *et al.*,^5,8^ Bakker *et al.*,^13^ and Maschmeyer *et al.*,^14,15^ with the hydrogenation of benzonitrile featuring prominently. More recently, Dai and co-workers have examined the application of supported Pd catalysts for the reduction of benzonitrile to benzylamine; considering a role for the support material, reactor configuration and catalyst deactivation in catalytic performance.^16–18^ Keane and co-workers have investigated the gas phase variant of this reaction.^19^ Illustrating imaginative exploitation of this technology, Saito and co-workers have applied a supported Pd catalyst operating in a continuous flow regime to employ a selective nitrile reduction stage to synthesize the antidepressant drug venlafaxine.^20^

In 2016 McMillan and co-workers reported on the hydrogenation of aromatic nitrile compounds over a carbon-supported Pd catalyst.^21^ Hydrogenation of benzonitrile to form benzylamine was readily achieved but this was subsequently followed by a hydrogenolysis reaction to produce toluene. Co-adsorption studies of benzonitrile and benzylamine provided evidence for site-selective chemistry over this particular Pd/C catalyst, with specific sites associated with the hydrogenation and hydrogenolysis pathways.

Connected to the background outlined above, an important parameter for reduction reactions incorporated within fine chemical synthesis regimes is to be able to better understand factors that contribute to the presence (or not) of hydrogenation and hydrogenolysis pathways. The reagent selected for the studies reported here is 4-hydroxybenzyl cyanide (HOC_6_H_5_CH_2_CN), which was selected as a reagent for two reasons. Firstly, selective hydrogenation of 4-hydroxybenzyl cyanide will yield 4-(2-aminoethyl)phenol (HOC_6_H_5_CH_2_CH_2_NH_2_), also known as tyramine and previously identified as a biologically valuable species. Secondly, this molecule is also found to be of relevance to certain agri-chemical production chains where selective nitrile hydrogenation is often required in the presence of other ring substituents. To a degree, the inclusion of the hydroxyl group para to the nitrile chain addresses this requirement.

Chemically, following on from the early work of Buck using a platinum oxide catalyst,^22^ there are surprisingly few accounts for the synthesis of this trace amine.^23–25^ Thus, this work seeks to use a potential synthesis route to a biologically relevant primary amine to additionally explore the factors that influence hydrogenation and hydrogenolysis steps over the same carbon-supported Pd catalyst used by McMillan *et al.*^21^ In this way, the selected reaction system will provide new information on the correlation between molecular structure and selectivity issues connected with the hydrogenation of substituted phenethylamines.

**Scheme 1:**
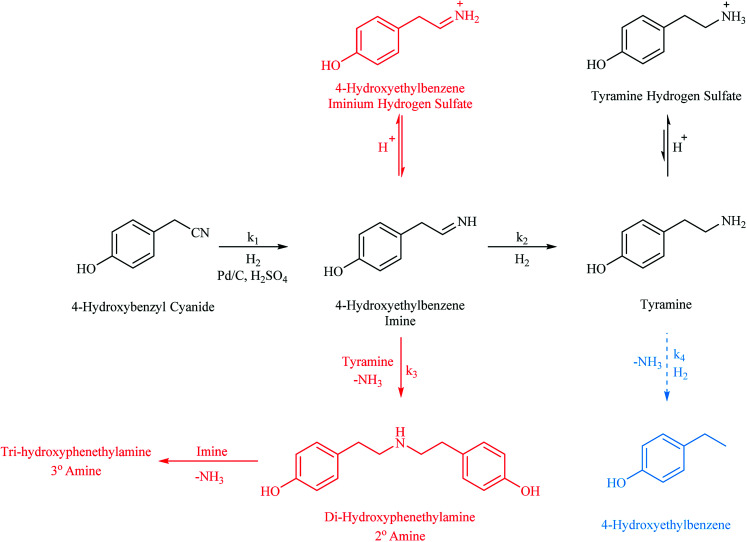
Reaction scheme corresponding to the hydrogenation of 4-hydroxybenzyl cyanide (*ca.* 17 mmol including *ca.* 34 mmol H_2_SO_4_) in MeOH (350 mL) over 0.50 g 5% Pd/C. The desired pathway is highlighted in black with unwanted routes, hydrogenolysis and coupling reactions, represented in blue and red respectively.

With reference to Scheme 1, the routes to the abovementioned selectivity issues associated with nitrile hydrogenations are outlined alongside the desired pathway. Notably, the potential hydrogenolysis, as observed for the benzonitrile system reported by McMillan,^21^ is shown. In this case, 4-hydroxyethylbenzene is expected upon loss of the amine functionality. In addition, highlighted in red and commonly observed in nitrile reductions, is the coupling reaction of the highly reactive imine intermediate, 4-hydroxybenzyl imine, with the amine products.^5,8^ Di-hydroxyphenylamine is formed by the coupling of this imine with tyramine in a reaction that is accompanied by the elimination of ammonia. It is then possible for the 4-hydroxybenzyl imine to couple with this secondary amine in a similar process to yield the tertiary amine tri-hydroxyphenylamine. The production of secondary and tertiary amines is a significant compromise to primary amine selectivity and highlights the essential nature of an adequate hydrogen supply which is shown to be essential in the production of tyramine (see Section 3.2).

Care was taken to explore the various experimental parameters that could potentially impede product formation. Consequently, invoking an experimental protocol that reduces the production of secondary and tertiary amines is essential in order to enhance selectivity towards the primary amine. Several authors report the use of auxillary agents to enhance primary amine yields. Amongst other methods, both acids (*e.g.* HCl),^26–28^ and bases (*e.g.* NH_3_),^29^ have been used for such a purpose.

On the assumption that protonation of the amine will reduce its reactivity as a nucleophile, and thus, prevent any coupling reactions, inclusion of an acid additive was implemented.^8^ In this instance, sulphuric acid was selected; an excess of a diprotic acid should ensure protonation of the amine functionality throughout the full reaction coordinate and, moreover, the anion (HSO_4_^−^) is not expected to cause metal complexation problems as might be the case if, for example, HCl was used. Furthermore, the protonation of the amine to form a salt should keep this species in the liquid phase, and in doing so, prevent its coordination to the active sites of the catalyst which could be of detriment to the conversion of the nitrile/imine.^8^ The strong chemisorption of the nitrogen lone pair to the Pd surface is therefore thwarted by the acid's presence and, as such, poisoning of the catalyst should be prevented. In order to achieve the selective production of the primary amine it is essential that the previously discussed hydrogenolysis and coupling reactions are eliminated from the system. These matters are the focus of this communication.

## 2. Experimental

Reactions were carried out in the liquid phase in a continuously stirred batch slurry reactor (500 mL, Büchi bmd) using methanol (Sigma Aldrich, ≥99.7%) as a solvent. An automated gas flow controller (BPC 1202) allowed the delivery of inert (N_2_, BOC, 99.999% purity) and active (H_2_, BOC, ≥99.995% purity) gases to be delivered directly to the reactor *via* a gas reservoir. Reactions were heated by silicon oil passed around the reactor *via* a heating circulator (Julabo F25). 4-Hydroxybenzyl cyanide (TCI, purity > 99%) was hydrogenated over a 5 wt% Pd/C catalyst (Sigma Aldrich, code number 205680) in the presence of a sulphuric acid additive (Sigma Aldrich, 99.999% purity). Characterisation of this catalyst, which was also used in the previously mentioned benzonitrile studies, has been undertaken elsewhere.^21^ Briefly, CO chemisorption determined the number of surface metal sites to be 1.11 × 10^20^ Pd_(s)_ atoms per g_(cat)_, corresponding to a Pd dispersion of 39%. Transmission electron microscopy revealed a mean Pd particle size of 2.5 nm.^21^

The reactor was charged with catalyst and solvent. The mixture was then heated and reduced under hydrogen for 1 hour prior to substrate addition *via* a syringe. The reaction mixture was sampled at regular intervals and analysed by HPLC (HP series 1100 HPLC fitted with a BDS Hypersil C18 column). In the resultant reaction profiles *t* = 0 is considered as the point where there is no reagent consumption or product formation. Experiments connected with high product yields were additionally analysed by ^1^H NMR spectroscopy using a Bruker AVI 400 MHz spectrometer. Inclusion of a known concentration of ethylene glycol in the NMR tube was used as an internal standard, which allowed quantification of both reagent and product. Deuterium oxide, selected for its effective peak resolution in this system, was utilised as the deuterated solvent for NMR analysis.

## 3. Results and discussion

### 3.1 Hydrogenation *versus* hydrogenolysis

Considering first the hydrogenolysis pathway, found to be accessible over this catalyst for the benzonitrile system, but also a phenomenon observed by other authors.^5,8,13,14,21^ Indeed, for benzonitrile hydrogenation over the same Pd/C catalyst, used in this instance for 4-hydroxybenzyl cyanide, hydrogenolysis was a dominant pathway to such a degree that, ultimately, toluene was produced with complete selectivity.^21^ Despite the incidence of hydrogenolytic cleavage of the amine group described in the case of benzonitrile, no 4-hydroxethylbenzene was detected in the liquid phase for the hydrogenation of 4-hydroxybenzyl cyanide (see Section 3.2).

Given that this is exactly the same Pd/C catalyst and experimental apparatus as used by McMillan *et al.*,^21^ the inability of this catalyst to support the proposed hydrogenolysis stage may initially be considered a surprising result. For the benzonitrile system the documented reactivity was interpreted within a 3-site model for the catalyst. Namely, one site was assigned to the hydrogenation of the nitrile/imine, whilst another facilitated the hydrogenolysis, a final site was then allocated for the dissociative adsorption of dihydrogen (which may also encompass the other sites). This model was supported by kinetic and co-adsorption studies. Thus, given that a hydrogenolysis function is known to occur on this catalyst, why is the hydrogenolysis stage inaccessible under any of the presented reaction conditions?

**Fig. 1:**
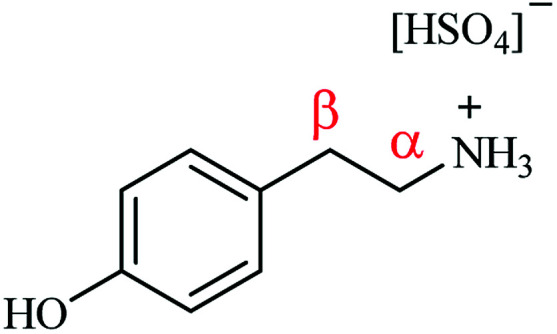
The molecular structure of the tyramine hydrogen sulfate salt. The α and β methylene units of the substituent arm are referenced relative to the amine functionality.

Fig. 1 presents the molecular structure of tyramine hydrogen sulfate and identifies the aliphatic carbon atoms as α and β positions relative to the amine functionality. It is suggested that it is the positioning of the α-carbon relative to the aromatic ring which dictates whether a hydrogenolysis pathway is supported over this catalyst. Indeed, Maschmeyer *et al.* similarly propose that it is the presence of an aromatic group adjacent to the α-carbon that facilitates hydrogenolysis.^14^ It is thus advocated that the ammonium leaving group needs to be connected to the aromatic ring *via* a single methylene group in order to facilitate the hydrogenolysis step. In the case of the tyramine hydrogen sulfate salt, the additional methylene linker in the substituent arm does not meet this molecular requirement, endorsing the absence of 4-hydroxyethylbenzene in the reaction mixture. This scenario implies that the site-selective hydrogenation/hydrogenolysis chemistry invoked by McMillan *et al.*^21^ is not a dominant process. Further, computational calculations conducted by Kieboom *et al.* on the hydrogenolysis of benzyl alcohol derivatives over Pd/C suggest that the aromatic ring is π-bonded to the catalyst surface. Here it is postulated that for hydrogenolysis to occur it is essential that the transition state must facilitate overlap between the electron deficient p-orbital of the α-carbon and the π-orbitals of the benzene ring. The necessity for this orbital overlap rationalises, on account of geometry, why an additional methylene linker, when compared to benzonitrile, between the aromatic group and the heteroatom is not conducive to a hydrogenolytic process.^30^

### 3.2 Towards the selective production of tyramine

**Fig. 2:**
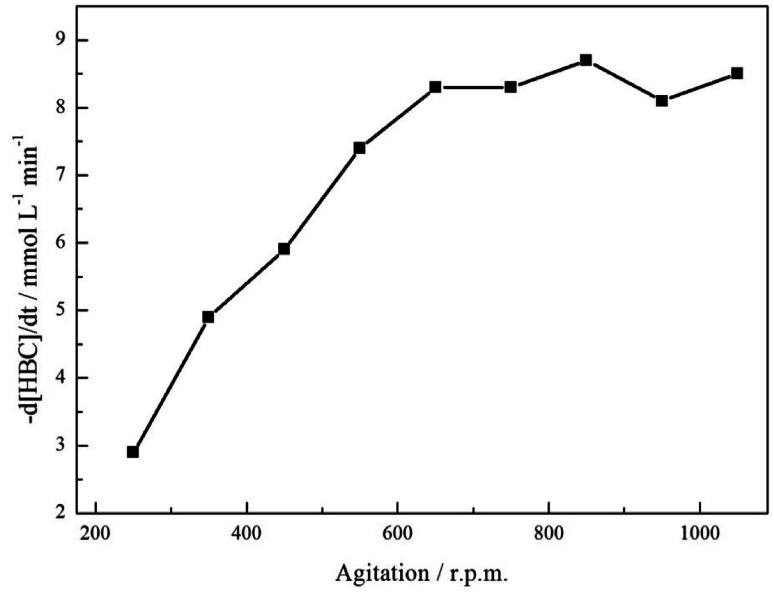
Influence of agitation rate on the hydrogenation of 4-hydroxybenzyl cyanide (*ca.* 17 mmol) over 0.50 g of a 5% Pd/C catalyst in the presence of 2 molar equivalents of acid. Reaction carried out at 60 °C and at 5 bar g pressure.

Section 3.1 states that a hydrogenolytic pathway is not operational for this substrate and, consequently, can be removed from consideration. Unfortunately, coupling reactions were found to complicate the reaction profile with an acid additive found to be essential for production of the primary amine. Inclusion of acid allowed complete removal of a tertiary amine component indicating that the additive was effective in hindering coupling reactions. Nonetheless, the route to the secondary amine remained accessible, restricting complete selectivity to the primary amine. The identification of the secondary and tertiary amines was achieved by LC-MS analysis and can be found in the ESI, S1.† Hydrogen availability in liquid phase nitrile hydrogenation reactions is a further critical experimental parameter that can provide a means for altering the selectivity of a reaction.^6^ Therefore, in order to evaluate the possibility of mass transport restrictions in the reaction of interest, the effect of agitation rate on the rate of consumption of the starting material was considered,^31^Fig. 2.

Fig. 2 shows the expected ‘plateau’ dependence of reagent consumption as a function of agitation rate at high mixing speeds. Over the range 650–1050 rpm, increasing stirring speeds leads to no real increase in the 4-hydroxybenzyl cyanide hydrogenation rate, indicative of the reaction being under kinetic control and free of mass transport restrictions. However, below *ca.* 650 rpm the reaction is exhibiting characteristics of mass transport limitations (reaction rate ∝ stirring speed) and is operating under diffusion control; presumably as a result of inefficient mass transport at the gas/liquid interface. Given this knowledge, and the inference from Scheme 1 that hydrogen supply is pivotal to tyramine formation, it is crucial that stirring is sufficiently fast as to avoid operation within a domain of constrained hydrogen supply to the Pd surface.

**Fig. 3:**
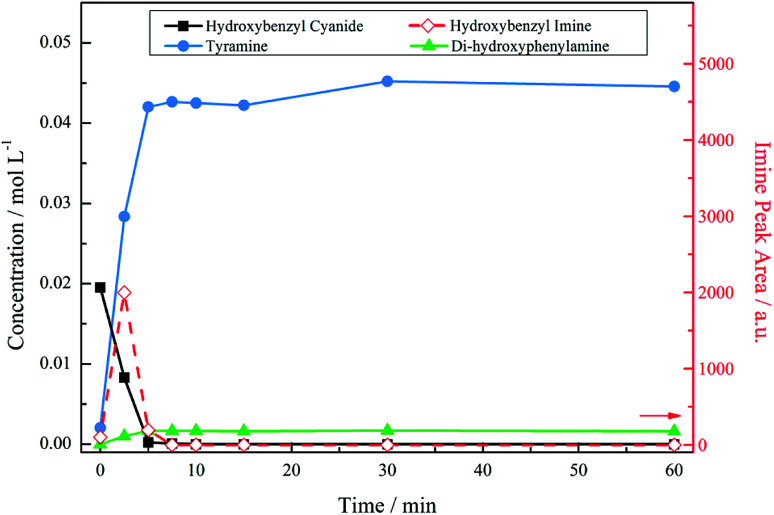
Reaction profile for 4-hydroxybenzyl cyanide hydrogenation under acidic conditions at an agitation rate of 1050 rpm [5 bar g, 60 °C, 0.50 g 5% Pd/C, *ca.* 17 mmol of nitrile, *ca.* 34 mmol H_2_SO_4_, 350 mL MeOH].

Fig. 3 presents the reaction profile for an acidic mixture operating at a stirring speed of 1050 rpm. Complete conversion of the starting material was achieved in approximately 5 minutes, significantly faster that the reaction in the absence of acid (*cf.* 93% conversion over 6 hours, not shown). The imine intermediate was rapidly consumed affording the primary amine tyramine with 92% selectivity. A complete mass balance was achieved by reaction completion with the remainder made up by the secondary amine formed *via* the aforementioned coupling reaction. It should be noted that Fig. 3 shows a concentration profile for the secondary amine. This species was not commercially available meaning that calibration by HPLC, as was employed for 4-hydroxybenzyl cyanide and tyramine, could not be achieved. Therefore, ^1^H NMR spectroscopy was used as a means of calculating a response factor based on the relative integration of peak areas allowing a concentration to be obtained. As a consequence of the highly reactive nature of imines, however, it was not possible to determine the response factor for the 4-hydroxybenzyl imine intermediate. As such, imine peak area was plotted seperately on the second *y*-axis. This is also the case for all future figures involving the imine species. The unsuccessful quantification of the imine results in an apparent missing mass at the beginning of the reaction. Nonetheless, a complete mass balance is returned by reaction completion, indicating the absence of undesirable side reactions (see ESI, S2†).

Despite the high selectivity towards tyramine achieved in Fig. 3, tuning of the reaction conditions and parameters was essential for this outcome to be realised. A brief summary of the optimisation process and the subsequent effect on selectivity is presented in Table 1. The most notable improvement was detected upon inclusion of an acid additive. The addition of this species to the reaction mixture effectively removed the tertiary amine from the system and drastically enhanced tyramine production. Further, at low stirring speeds, found to be outside the kinetic regime for this system (Fig. 2), the hydrogen supply was found to be limited resulting in an unoptimised selectivity. This can be evidenced by the marked improvement to tyramine production (82% → 92%) upon elevation of agitation rate. Thus, the combination of employing an acid as an auxiliary agent plus operation within a kinetic regime effectively free from gas/liquid mass transport limitations has led to significant improvements in the returnable yield of the primary amine salt (tyramine hydrogen sulfate). These alterations illustrate the intricate nature of the process involved in achieving the completely selective production of a primary amine.

**Table 1:** Selectivity changes observed as a result of various parameter alterations. All values are calculated at 60 minutes into the reaction. In all instances the following were constant: [5 bar g, 0.50 g 5% Pd/C, *ca.* 17 mmol of nitrile, *ca.* 34 mmol H_2_SO_4_, 350 mL MeOH]. For all reactions involving acid it is the salt of the amine that is detected. *Only 93% conversion achieved over 6 hours

Conditions			Selectivity		
Acid	*S*/rpm	*T*/°C	1° amine/%	2° amine/%	3° amine/%
*No	450	60	14	44	30
Yes	450	60	82	18	0
Yes	1050	60	92	8	0
Yes	1050	30	100	0	0

It should be noted that the reaction parameters presented here do not represent an exhaustive list of factors influential over hydrogen supply. Additionally considered were the effects of hydrogen pressure in the headspace of the autoclave. Increasing hydrogen pressure resulted in faster hydrogenation rates; notably complete conversion of 4-hydroxybenzyl cyanide was attained at 10, 5 and 2.5 minutes at respective overpressures of 3, 5, and 7 bar g. Outwith the error bars associated with the analytical measurement, a sensitivity of selectivity appears to be apparent. Namely, increasing the operational pressure from 3 to 5 bar g increases tyramine hydrogen sulphate selectivity on completion of reaction from ∼81% to ∼94%. A further increase in pressure to 7 bar g, however, was found to be approximately equal to the 5 bar g reaction, indicating that no further improvements could be made to selectivity *via* pressure within the limitations of the experimental set-up.

Nevertheless, the necessity to improve selectivity towards the primary amine remained. Thus, in order to further improve selectivity to the primary amine, the role of temperature was explored. Intriguingly, the literature provides conflicting opinions on the effect of temperature on nitrile hydrogenation reactions. Augustine, for example, suggests that high temperatures favour formation of primary amines as a consequence of desorption of the imine intermediate from the surface being minimised under these conditions.^32^ In contrast, Cerveny indicates that it is secondary amine formation that is favoured by elevated temperatures.^33^ Clearly, temperature also links to the hydrogen availability issues central to tyramine formation. Arrhenius theory tells us that increasing temperature can increase reaction rates but, given the breadth of pathways accessible to this reaction system presented in Scheme 1, it is desirable to disfavour the rate for the detrimental coupling reactions. Further interwoven into the complexity of the reaction matrix is the matter of hydrogen solubility in the process solvent: hydrogen solubility in methanol is enhanced at elevated temperatures.^31^ It is therefore necessary to identify an optimal temperature that will facilitate effective solubility of hydrogen in methanol, whilst additionally maintaining the selectivity of the reaction.

**Fig. 4:**
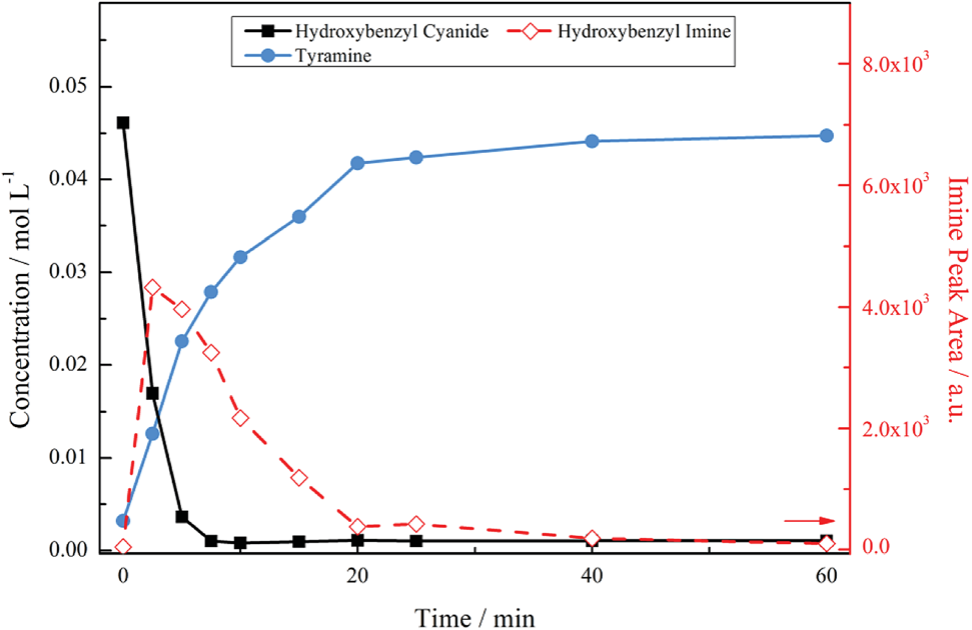
Reaction profile for 4-hydroxybenzyl cyanide hydrogenation under acidic conditions carried out at a temperature of 30 °C [5 bar g, 1050 rpm, 0.50 g 5% Pd/C, *ca.* 17 mmol of nitrile, *ca.* 34 mmol H_2_SO_4_, 350 mL MeOH].

Temperature alterations on the system were implemented, with Fig. 4 showing the reaction profile for 4-hydroxybenzyl cyanide hydrogenation under acidic conditions at 30 °C. The decrease in temperature had a significant effect on the product distribution. As before, the nitrile unit is rapidly consumed with complete conversion to form the tyramine salt *via* the 4-hydroxybenzyl imine intermediate. However, no secondary or tertiary amine products are identified chromatographically in this case, indicating that the primary amine has been selectivity produced. Thus, Fig. 4 represents a totally selective conversion of 4-hydroxybenzyl cyanide in an acidic medium to form tyramine hydrogen sulphate in the liquid phase.

With reference to Scheme 1, the selectivity of the reaction (*S*) can be expressed in terms of the kinetics of primary amine formation *versus* the coupling reactions to form the secondary and tertiary reactions as *S* = *k*_2_ [H_2_]/*k*_3_[primary amine]. Lowering the reaction temperature will affect both of the rate constants presented here and the improved selectivity evident in Fig. 4 is reflective of the difference in the activation energies of these competing reactions. A lower activation energy for the hydrogenation reaction to the primary amine than for the coupling reactions could provide reasoning for the improved selectivity at lower temperatures. Another explanation, however, may be formulated. Namely, that if the hydrogen supply in the system has been optimised to avoid gas/liquid mass transport limitations, thus allowing a fixed concentration of hydrogen in solution, it is also possible that a liquid/surface mass transport limitation could occur. In this particular case the hydrogen availability for the high temperature reaction is lower and is detrimental for selectivity. Further work is required for these two hypotheses to be differentiated.

The reaction profile exhibits the form of a consecutive reaction. 4-Hydroxybenzyl cyanide conversion is rapid, being complete within a reaction time of ∼8 minutes; the complete conversion of 4-hydroxybenzyl cyanide corresponds to a turnover number of 184. Hydrogenation of the hydroxybenzyl imine to produce tyramine is clearly a much slower process. The product is formed on commencement of reaction and progressively increases thereafter; the reaction is complete by ∼40 minutes. This sequence indicates the imine hydrogenation step to be rate limiting under the designated reaction conditions.

**Fig. 5:**
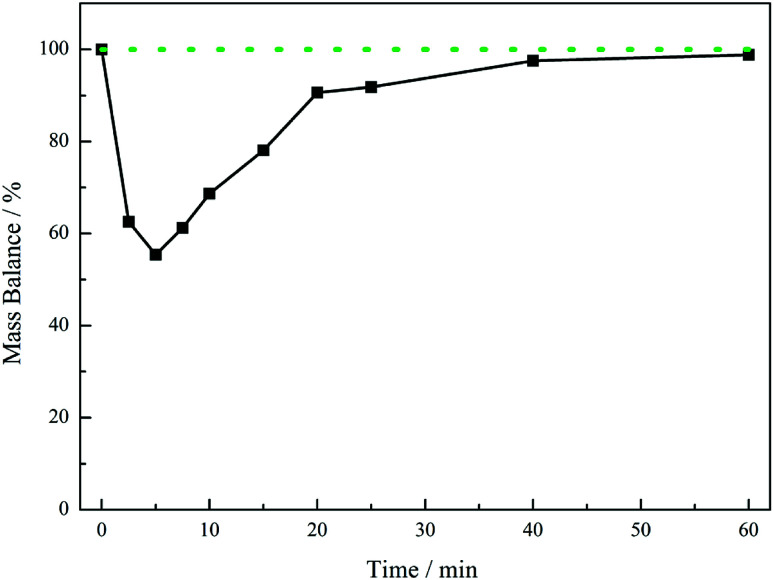
Mass balance plot for the reaction profile presented in Fig. 4 (4-hydroxybenzylcyanide hydrogenation, 30 °C, 5 bar g, 1050 rpm, 0.50 g 5% Pd/C, *ca.* 17 mmol of nitrile, *ca.* 34 mmol H_2_SO_4_, 350 mL MeOH). The solid black line defines the experimental mass balance (derived from quantification of hydroxybenzyl cyanide and tyramine, Fig. 4), whereas the dotted green line represents the theoretical mass balance.

Fig. 5 presents the mass balance plot for the reaction profile presented in Fig. 4. Upon commencement of reaction there is a clear mass imbalance over the period 0–20 minutes. With reference to Fig. 4, this corresponds to the period in which chromatography detects the presence of the 4-hydroxybenzyl iminium hydrogen sulphate in the liquid phase. From approximately 12 minutes the mass balance loss progressively reduces, so that by approximately 40 minutes a closed mass balance is obtained. Collectively Fig. 4 and 5 indicate that during the earlier stages of the reaction coordinate the 4-hydroxybenzyl imine is responsible for the mass imbalance.

An additional point worthwhile noting relates to the possibility of homogeneous reactions occurring within this reaction system. The reaction profile depicted in Fig. 4 shows a total absence of secondary and tertiary amine by-products after 60 minutes reaction time. However, when sample vials containing the colourless analyte were left for an extended period of time (overnight) under ambient conditions, certain samples exhibited a distinct colour change. Specifically, those samples that contain both the 4-hydroxybenzyl iminium hydrogen sulfate and the tyramine hydrogen sulfate (*i.e.* samples corresponding to the reaction period 5–15 minutes) developed a brown colouration whilst, in marked contrast, samples connected with reaction times where the imine was absent (*i.e.* 20–60 minutes) remained colourless. The colouration is assumed to reflect homogeneous chemistry that results in the formation of higher molecular weight conjugated molecules from a reaction between solvated 4-hydroxybenzyl iminium hydrogen sulfate and the tyramine hydrogen sulfate entities. This action highlights the reactive nature of the imine species. Nonetheless, given the relatively short reaction time of the heterogeneous chemistry illustrated in Fig. 4 (≤60 minutes) compared to the long period of the homogeneous process (≥12 hours), the observed homogeneous chemistry is not a major concern for the heterogeneously catalysed selective production of tyramine under consideration here.

**Fig. 6:**
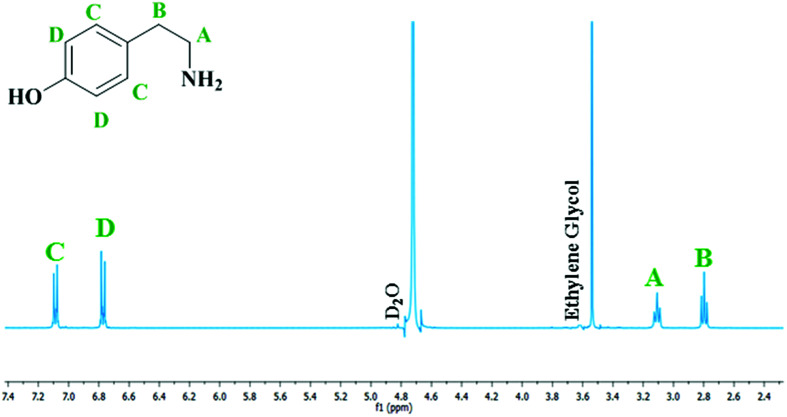
Resultant ^1^H NMR spectrum at reaction completion (*t* = 60 minutes) for the hydrogenation of 4-hydroxybenzyl cyanide to tyramine hydrogen sulfate at 5 bar g pressure, 30 °C and an agitation speed of 1050 rpm. The D_2_O peak of the NMR solvent is present at *δ* ∼ 4.9 ppm, whilst the ethylene glycol reference peak is present at *δ* = 3.65 ppm.

The mass imbalance detected by HPLC, despite being transient and its presence rationalised, is less than ideal. Nevertheless, whilst the primary analytical technique used to examine this molecular system was liquid chromatography, proton NMR spectroscopy was also found to be a suitable probe to monitor the progress of the reaction. Fig. 6 therefore shows a representative ^1^H NMR spectrum at reaction completion (*t* = 60 minutes) corresponding to the optimised reaction conditions utilised to afford Fig. 4. Spectra taken throughout the reaction coordinate along with complete spectral assignment and the corresponding reaction profile can be found in the ESI (S3†).

Analysis of the spectra presented in Fig. 6 reveals that, with the exception of features connected with the NMR solvent (D_2_O) and the internal standard (ethylene glycol), only peaks associated with tyramine hydrogen sulphate are present.^34^ No other features were detected. Thus, at reaction completion Fig. 6 endorses the outcome of Fig. 4; namely, the selective formation of the primary aromatic amine hydrogen sulfate salt from the catalytic hydrogenation of hydroxybenzyl cyanide.

Whilst the completely selective production of tyramine represents a satisfactory outcome, the batch conditions utilised do not sufficiently provide an evaluation of catalyst recyclability and durability towards poisoning. This issue is acknowledged to be one of specific relevance to this and related substrates and, consequently, catalyst deactivation investigations represent work in progress.

## 4. Conclusions

A Pd/C catalyst has been used to effect the hydrogenation of 4-hydroxybenzyl cyanide within a three-phase reactor to yield the primary amine tyramine. Alongside the desired pathway, additional routes, resulting in unwanted by-products, were identified. The absence of a hydrogenolytic cleavage rationalised by the unfavourable molecular framework of this species for hydrogenolysis preserved the valuable amine functionality. It was additionally found that coupling products, impairing the selectivity, could be effectively eliminated by tuning various experimental parameters leading to improved catalytic performance, such that the desired tyramine salt could be produced in quantitative yield.

## Conflicts of interest

There are no conflicts of interest.

## Supplementary Material

RA-008-C8RA05654D-s001
