# Supplementary material for: The production of tyramine via the selective hydrogenation of 4-hydroxybenzyl cyanide over a carbon-supported palladium catalyst
Source: RSC Adv. 2018 Aug 20;8(51):29392–9. doi: 10.1039/c8ra05654d (PMC9084560; doi:10.1039/c8ra05654d)
Supplement: RA-008-C8RA05654D-s001 [file RA-008-C8RA05654D-s001.pdf]

Electronic Supplementary Material (ESI) for RSC Advances.
This journal is © The Royal Society of Chemistry 2018

Supplementary Information

The production of tyramine via the selective hydrogenation of 4-hydroxybenzyl cyanide over a carbon-supported palladium catalyst

Mairi I. McAllister[a], Cédric Boulho[a], Liam McMillan[a], Lauren Gilpin[a], Sandra Wiedbrauk[a], Colin Brennan[b] and David Lennon*[a]

[a] School of Chemistry, Joseph Black Building, University of Glasgow, Glasgow, G12 8QQ, UK.

[b] Syngenta, Jeallot's Hill International Research Centre, Bracknell, Berkshire, RG42 6EY, UK.

S1. Chromatographic identification of secondary and tertiary amines observed in solution

S2. Mass balance in the presence of the imine intermediate for the hydrogenation of hydroxybenzyl cyanide reaction presented in Figure 3

S3. ^{1}H NMR Spectral Analysis under Optimised Reaction Conditions

S1. Chromatographic identification of secondary and tertiary amines observed in solution

Identification of the two unknown peaks present in the reaction profile presented in Figure 1 was carried out using the following analytical techniques. Liquid chromatography was initially used to determine the number of reaction components. GC/MS subsequently confirmed the presence of hydroxylbenzyl cyanide, whilst further analysis by LC/MS identified the remaining two unknowns. Two reaction samples that contained all three reaction intermediates underwent analysis. Samples were analysed as received without further dilution.

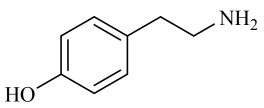

1° Amine
Tyramine
GFM = 137 g/mol

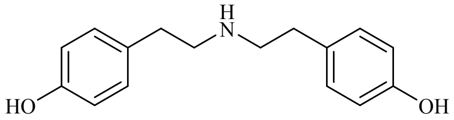

2° Amine
Di-hydroxyphenethylamine
GFM = 257 g/mol

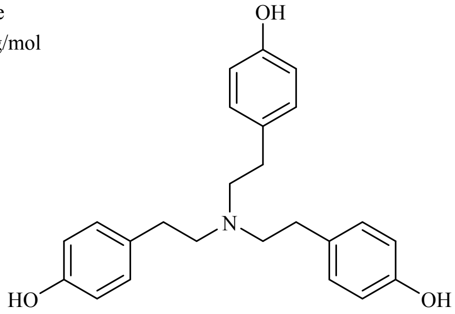

3° Amine
Tri-hydroxyphenethylamine
GFM = 377 g/mol

Results

Sample 1:

LC chromatogram

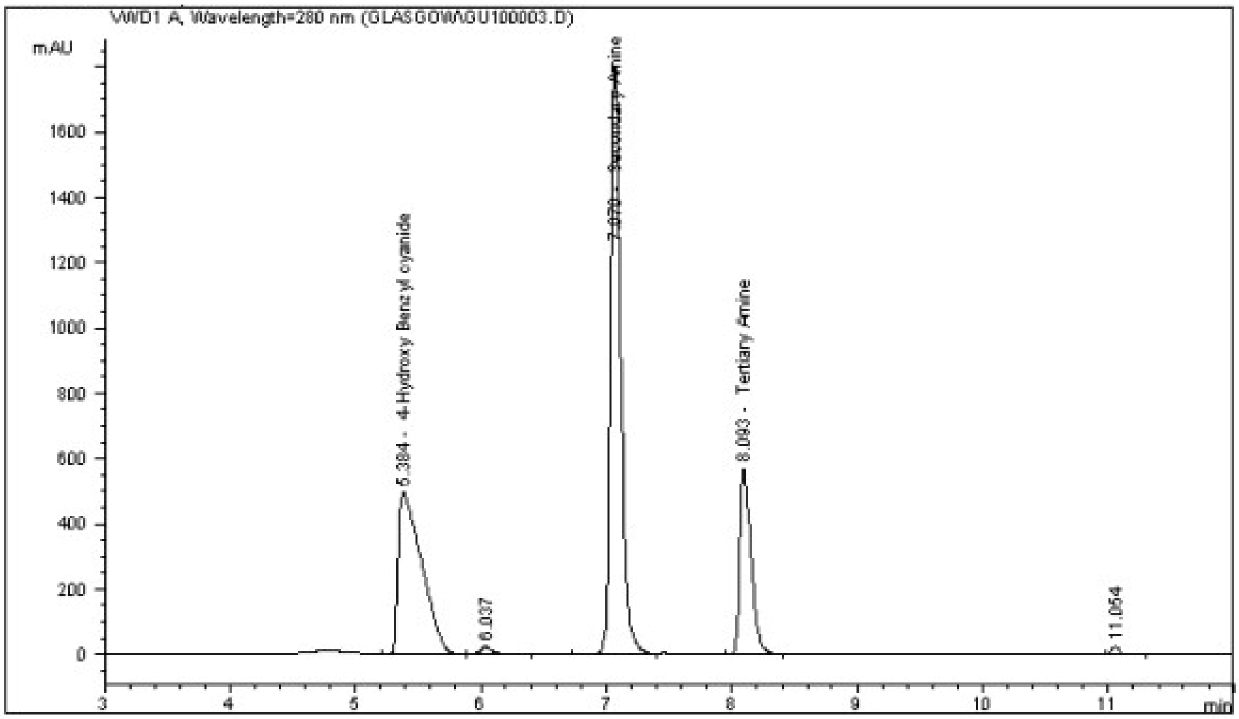

m/z

LC/MS analysis of peaks 2 and 3 (RT's 7.070 and 8.093 minutes) - secondary and tertiary amine

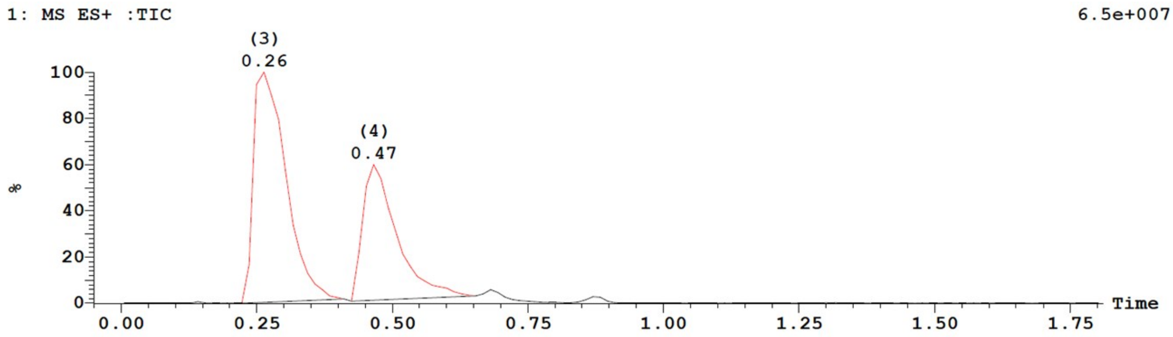

Peak ID Time
3 0.26

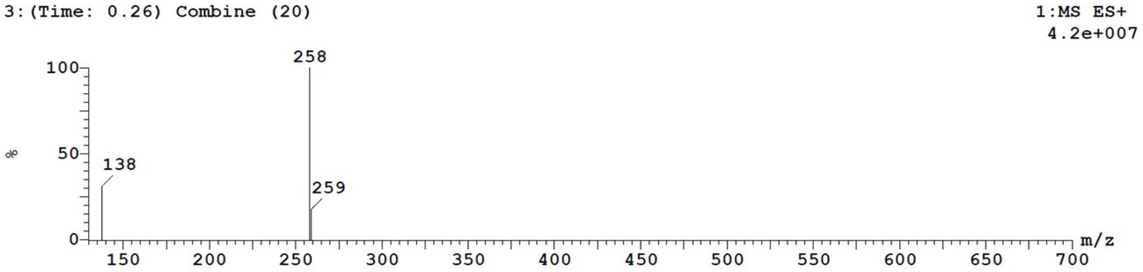

Peak ID Time
4 0.47

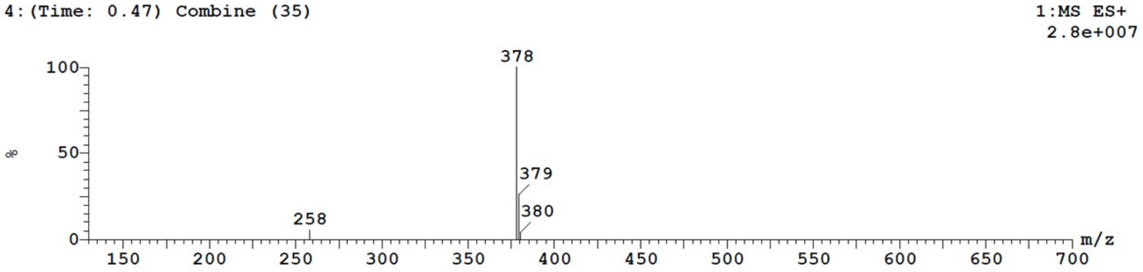

Sample 2:

LC chromatogram

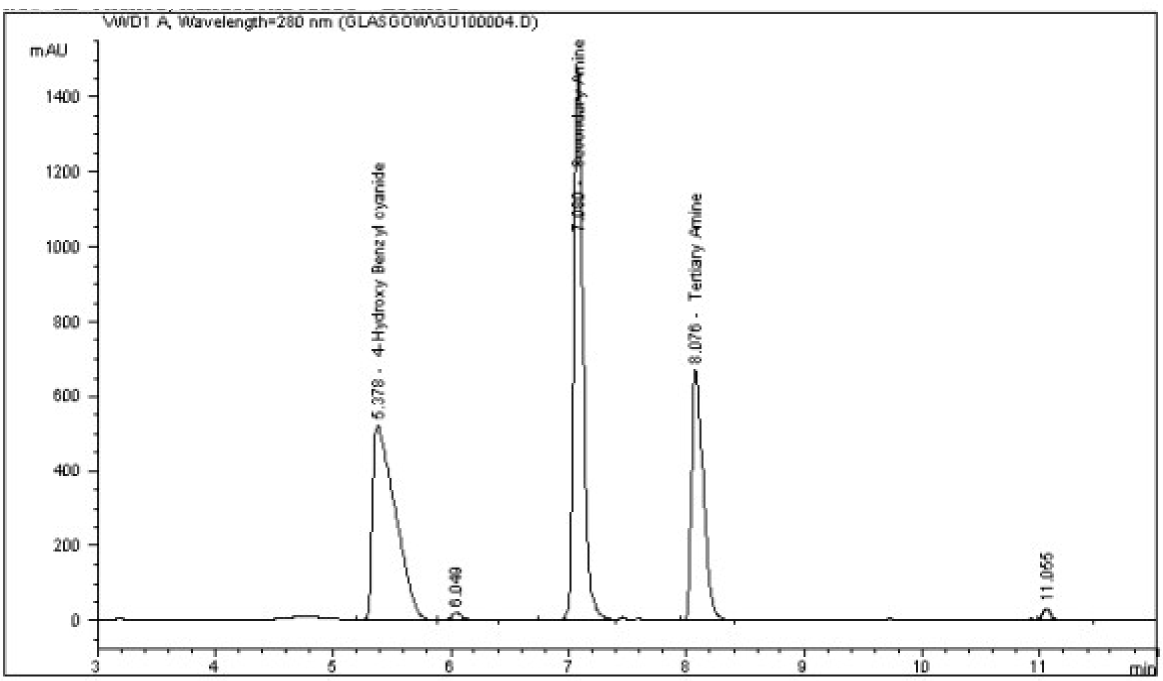

Peak 1 (RT = 5.378 minutes) 4-Hydroxybenzyl cyanide, as confirmed by GC/MS data above.

LC/MS analysis of peaks 2 and 3 (RT’s 7.080 and 8.076 minutes) - secondary and tertiary amine

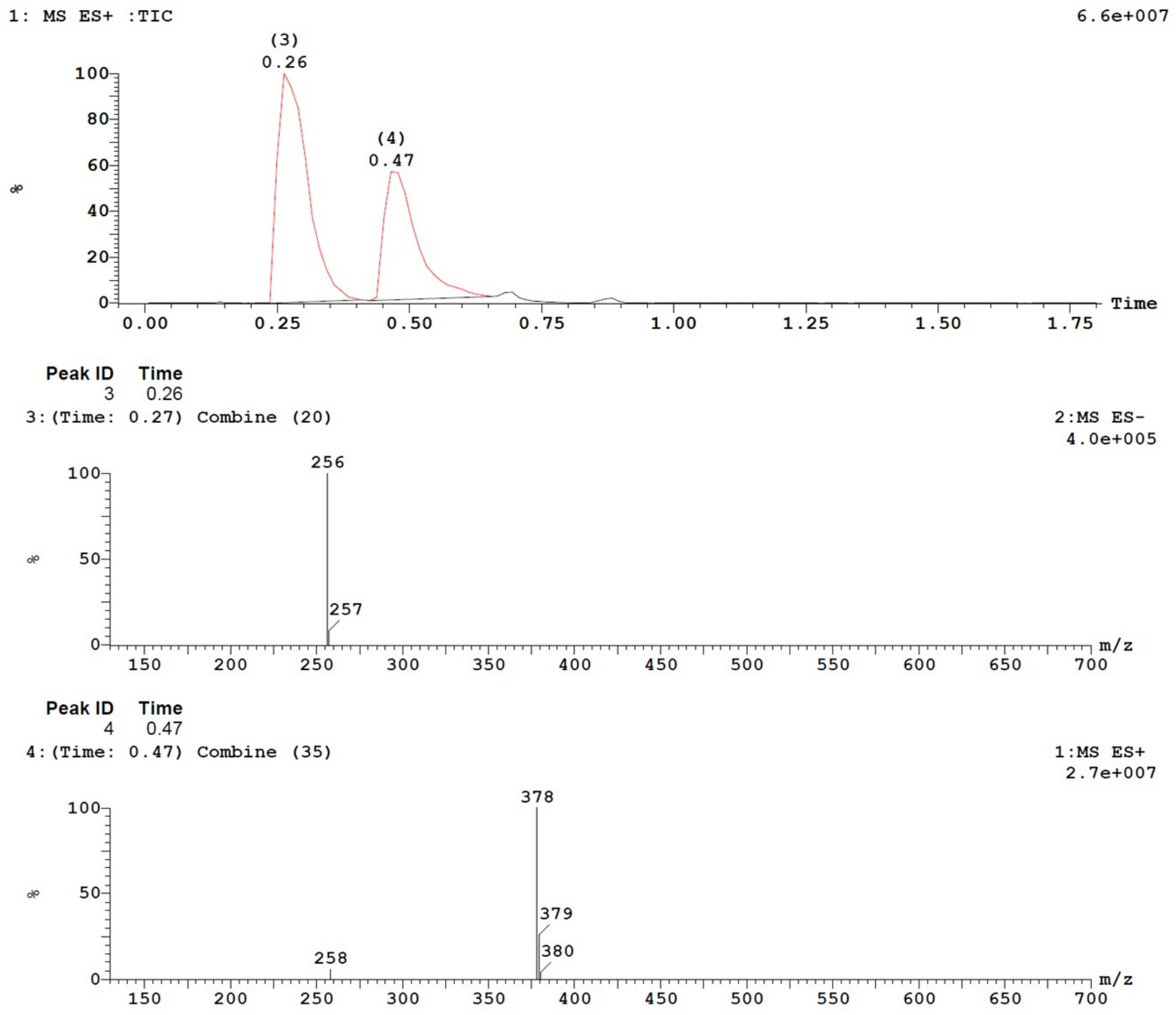

<u>Conclusions</u>

Authentic samples could not be obtained to give definitive identification. However, analysis by LC/MS provides the correct M^+ value when electrospray ionisation effects are taken into account. It is therefore proposed that the two unknowns with RT’s 11.5 and 14.9 are the secondary (di-hydroxyphenethylamine) and tertiary (tri-hydroxyphenethylamine) amines respectively.

Note: Positive electrospray (ES^+) gives [$M^{+\cdot}$ +H] and negative electrospray (ES^-) gives [$M^{+\cdot}$ – H].

Analytical Methods and Instruments

LC

Agilent 1100

Column: Zorbax Eclipse C18, 150 x 4.6 mm

Mobile phase: **A)** 0.1 % v/v TFA in water

B) 0.1% v/v TFA in Acetonitrile

Temperature: 25 °C

Flow: 1 mL min^{-1}

Gradient:

Time (min)	%A	%B
0	85	15
13	80	20
10	40	60
12	85	15
15	85	15

GC/MS

Shimaduz QP2010 plus

Column: Restek Rtx – 1 ms, 30 m, 0.25 mmID

Ramp: 1 min–4 min 25–300 °C

Time (min)	Temp (°C)
0	50
1	50
10	300

LC/MS

Waters Aquity UPLC

Column: xTerra MS C18 3.0 x 50 mm

Flow: 2.5 mL min^{-1}

Mobile phase: **A)** 0.1% formic acid

B) Acetonitrile

S2. Mass balance in the presence of the imine intermediate for the hydrogenation of hydroxybenzyl cyanide reaction presented in Figure 3

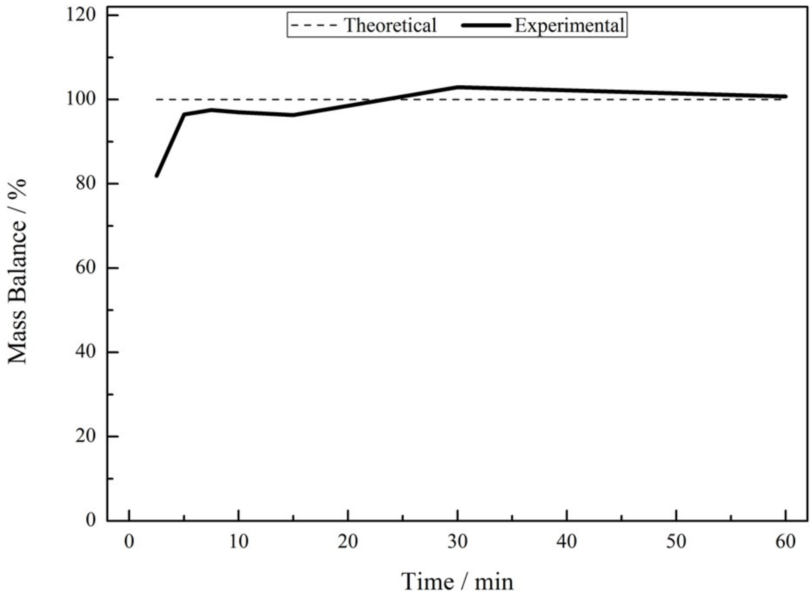

S2a. *Mass balance for the hydrogenation of hydroxybenzyl cyanide hydrogenation under conditions representing diffusion control as presented in Figure 3. The dashed line represents the calculated Ao with the solid line showing the experimentally determined vale.*

S2a shows the missing mass observed at the beginning of the reaction which is attributed to the presence of the uncalibrated imine intermediate. By the end of the reaction, where the imine was fully reacted, a complete mass balance was returned indicating that unwanted side reactions which would compromise selectivity are not in occurrence. In order to show this more clearly the reaction profile for the reaction at a stirring speed shown to be under diffusion control has also been included (**S2b**). The decreased rate of reaction allows the return of this mass balance upon complete reaction of the imine to be observed with greater clarity (**S2c**).

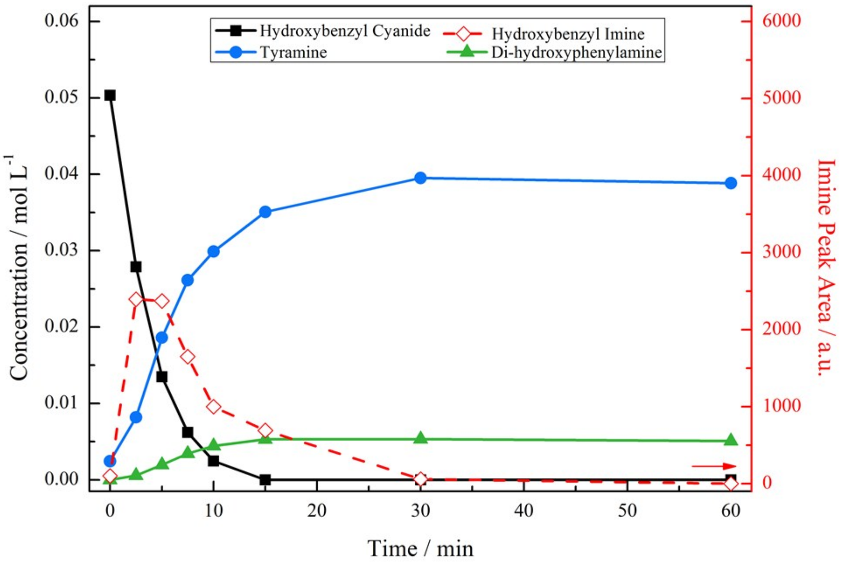

S2b *Reaction profile for 4-hydroxybenzyl cyanide hydrogenation under acidic conditions and diffusion control [5 barg, 60 °C, 450 rpm, 0.50 g 5% Pd/C, ca. 17 mmoles of nitrile, ca. 34 mmoles H_2SO_4, 350 mL MeOH].*

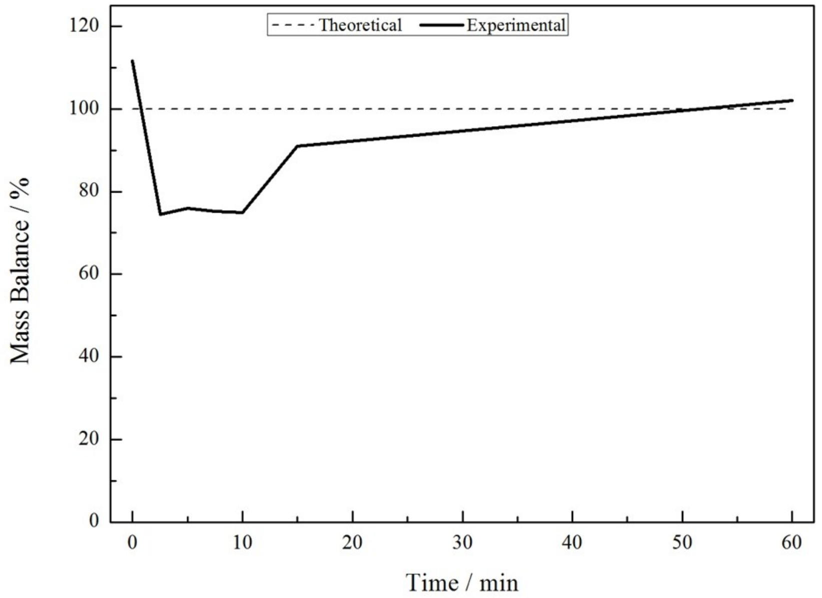

S2c. *Mass balance for the hydrogenation of hydroxybenzyl cyanide hydrogenation under conditions representing diffusion control as presented in **S2b**. The dashed line represents the calculated Ao with the solid line showing the experimentally determined vale.*

S3. ^{1}H NMR Spectral Analysis under Optimised Reaction Conditions

Figure S3a shows the ^{1}H NMR spectra corresponding to the reaction profile displayed in Figure 4 of the main text. For greater clarity, representative peaks for 4-hydroxybenzyl cyanide and tyramine have been boxed, highlighting their respective depletion and development as the reaction proceeds to completion. All of the chemical shifts are normalised to the residual methanol peak at 3.34 ppm. Nevertheless, due to the presence of acid in the reaction mixture, the signals exhibit a degree of frequency drift, most notably observed in the residual HDO signal at 4.79 ppm. Further, the imine species detectable by HPLC is not observed. This is a consequence of the practicalities of the NMR technique that required removal of methanol from the sample to allow spectra free from the reaction solvent to be obtained. The solvent evaporation stage was completed overnight and thus the inherent reactivity of the imine means that this species was too short lived to be present during analysis.

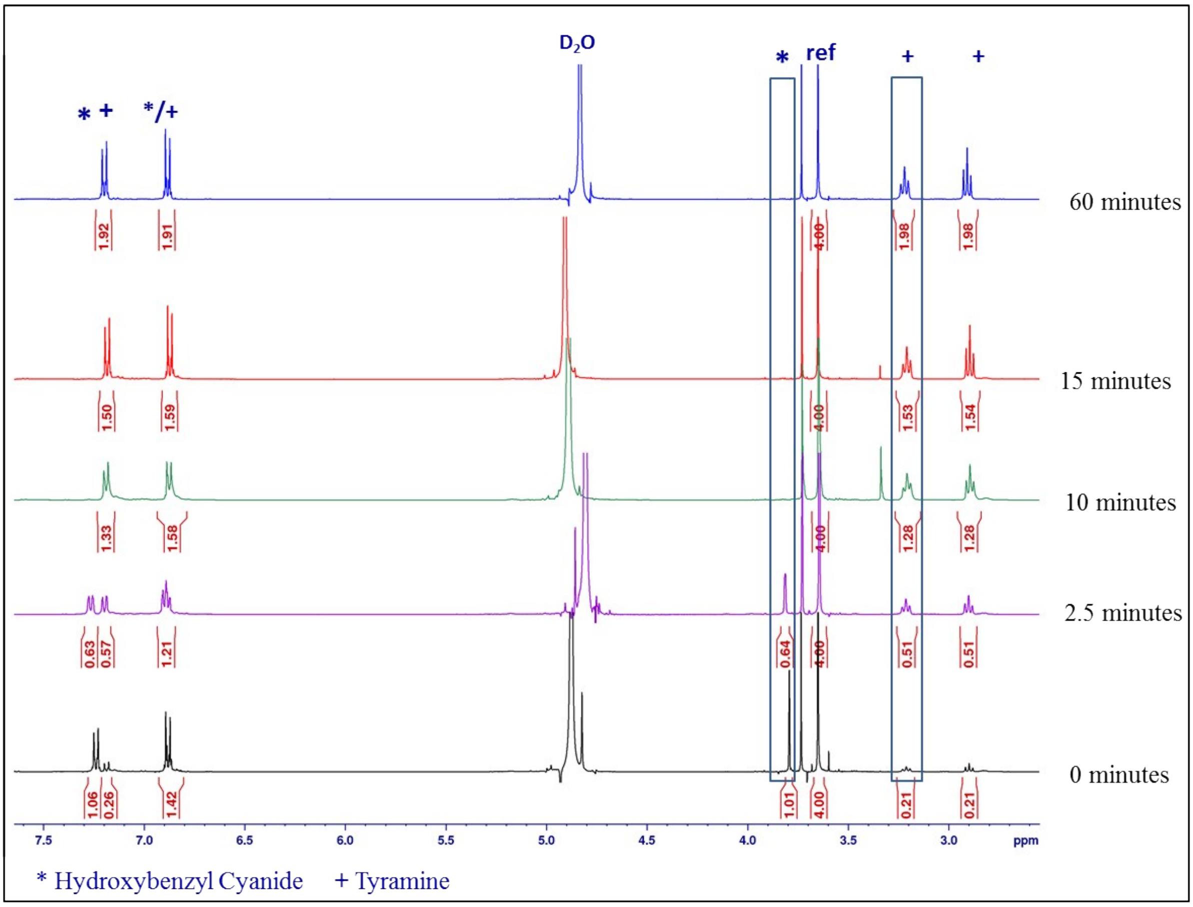

S3a. ^{1}H NMR spectra for the hydrogenation of 4-hydroxybenzyl cyanide to tyramine hydrogen sulfate at 5 barg pressure, 30°C and an agitation speed of 1050 rpm. Peaks representative of staring material (∗) and product (+) that have been selected for integration are indicated by boxes. The D_2O peak of the NMR solvent is present at $\delta \sim 4.9$ ppm, whilst the ethylene glycol reference peak is present at $\delta = 3.65$ ppm.

Integration of the highlighted reagent and product peaks referenced to the internal standard enables a NMR reaction profile to be produced, which is presented in S3b. The agreement between Figures 4 (main text) and S3b is evident. 4-Hydroxybenzyl cyanide is consumed within 10 minutes and tyramine production is complete at ~ 40 minutes reaction time. Moreover the mass balance profile of Figure 5 is essentially reproduced in Figure S3b, typified by an initial mass imbalance that slowly recovers over time to ultimately return a complete mass balance once hydrogen consumption has terminated. The eventual complete mass balance, along with the absence of unidentified products in the spectra

confirms that, despite the range of accessible pathways illustrated in Scheme 1, under the specified reaction conditions, the complete conversion of the starting material in to the desired product is indeed achievable.

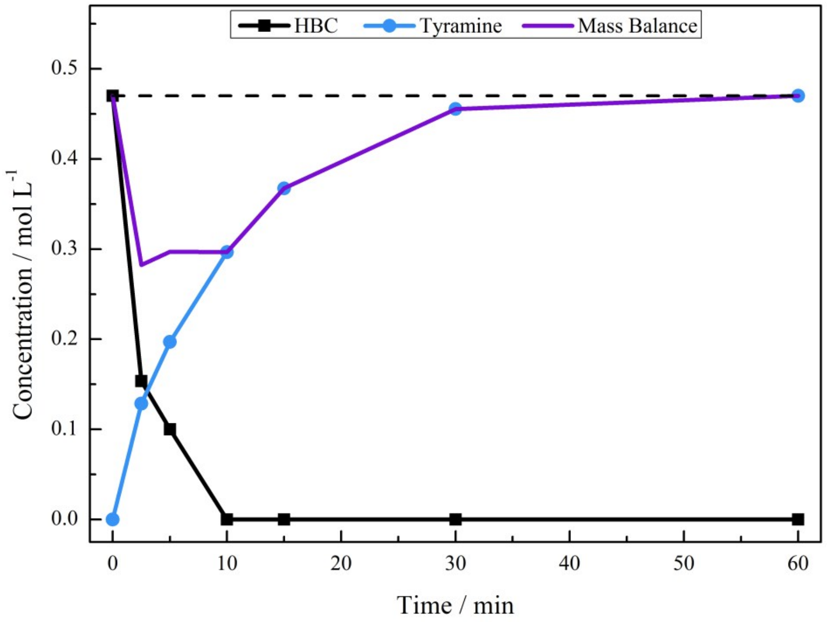

S3b. A reaction profile obtained from the series of ^{1}H NMR spectra presented in Figure 6 (the hydrogenation of 4-hydroxybenzyl cyanide to tyramine hydrogen sulfate at 5 barg pressure, 30°C and an agitation speed of 1050 rpm). Black squares define the concentration of 4-hydroxybenzyl cyanide, blue circles define the concentration of tyramine hydrogen sulphate. The solid purple line represents the experimental mass balance, whilst the dashed line represents the theoretical mass balance.

**Hydroxybenzyl Cyanide Assignment: t = 0 min**				
**Chemical Shift / ppm**	**Integration**	**Multiplicity**	**J-Coupling / Hz**	**Assignment**
3.69	2H	Singlet	-	CH_2benzyl
6.78	2H	Doublet	8.7	CHar
7.13	2H	Doublet	8.7	CHar

**Tyramine Salt Assignment: t = 60 min**				
**Chemical Shift /**	**Integration**	**Multiplicity**	**J-Coupling / Hz**	**Assignment**

ppm				
2.79	2H	Triplet	7.2	$CH_2\boldsymbol{CH_2}NH_2$
3.11	2H	Triplet	7.3	$\boldsymbol{CH_2}CH_2NH_2$
6.78	2H	Doublet	8.6	CHar
7.09	2H	Doublet	8.7	CHar

***S3.** ^{1}H NMR assignments for hydroxybenzyl cyanide (t = 0 min) and tyramine (t = 60 min). Samples were run in deuterium oxide using a 400 MHz spectrometer.*

Hydroxybenzyl Cyanide:
^{1}H NMR (D_2O, 400 MHz): 7.13 (2H, d, J = 8.7 Hz, CH_{ar}); 6.78 (2H, d, J = 8.7 Hz, CH_{ar}); 3.69 (2H, s, CH_2benzyl).

Tyramine Salt:
^{1}H NMR (D_2O, 400 MHz): 7.09 (2H, d, J = 8.7 Hz, CH_{ar}); 6.78 (2H, d, J = 8.6 Hz, CH_{ar}); 3.11 (2H, t, J = 7.3 Hz, $\boldsymbol{CH_2}CH_2NH_2$); 2.79 (2H, t, J = 7.2 Hz, $CH_2\boldsymbol{CH_2}NH_2$).
